# Effect of hypoxia on equine mesenchymal stem cells derived from bone marrow and adipose tissue

**DOI:** 10.1186/1746-6148-8-142

**Published:** 2012-08-22

**Authors:** Beatriz Ranera, Ana Rosa Remacha, Samuel Álvarez-Arguedas, Antonio Romero, Francisco José Vázquez, Pilar Zaragoza, Inmaculada Martín-Burriel, Clementina Rodellar

**Affiliations:** 1 Laboratorio de Genética Bioquímica (LAGENBIO), Facultad de Veterinaria, Universidad de Zaragoza, 50013, Zaragoza, Spain; 2 Hospital Veterinario, Facultad de Veterinaria, Universidad de Zaragoza, 50013, Zaragoza, Spain; 3 Instituto Aragonés de Ciencias de la Salud (IACS), Zaragoza, 50009, Spain

**Keywords:** Hypoxia, Horse, AT-MSC, BM-MSC, Characterisation

## Abstract

**Background:**

Mesenchymal stem cells (MSCs) derived from bone marrow (BM-MSCs) and adipose tissue (AT-MSCs) are being applied to equine cell therapy. The physiological environment in which MSCs reside is hypoxic and does not resemble the oxygen level typically used in *in vitro* culture (20% O_2_). This work compares the growth kinetics, viability, cell cycle, phenotype and expression of pluripotency markers in both equine BM-MSCs and AT-MSCs at 5% and 20% O_2_.

**Results:**

At the conclusion of culture, fewer BM-MSCs were obtained in hypoxia than in normoxia as a result of significantly reduced cell division. Hypoxic AT-MSCs proliferated less than normoxic AT-MSCs because of a significantly higher presence of non-viable cells during culture. Flow cytometry analysis revealed that the immunophenotype of both MSCs was maintained in both oxygen conditions. Gene expression analysis using RT-qPCR showed that statistically significant differences were only found for *CD49d* in BM-MSCs and *CD44* in AT-MSCs. Similar gene expression patterns were observed at both 5% and 20% O_2_ for the remaining surface markers. Equine MSCs expressed the embryonic markers *NANOG*, *OCT4* and *SOX2* in both oxygen conditions. Additionally, hypoxic cells tended to display higher expression, which might indicate that hypoxia retains equine MSCs in an undifferentiated state.

**Conclusions:**

Hypoxia attenuates the proliferative capacity of equine MSCs, but does not affect the phenotype and seems to keep them more undifferentiated than normoxic MSCs.

## Background

In recent years, mesenchymal stem cells (MSCs) have become increasingly utilised in regenerative medicine and tissue engineering applications because of their properties for self-renewal, differentiation and immunoregulation [1].

To study these properties, MSCs must be isolated from their physiological niches and cultured *ex vivo*. The micro-environment that cells experience in laboratory culture is very different from their native settings; therefore, it is possible that the true *in vivo* properties of these cells might be modified by artificial culture. One environmental property that is commonly altered by the change of environment is the percentage of oxygen. Traditional incubators are supplied with atmospheric air that contains 20% oxygen (defined as “normoxia”), which is a not physiologically accurate for any kind of cell. Two common MSC sources are bone marrow and adipose tissue, in which the oxygen tension ranges from 1%-7% [2] and 2%-8% [3], respectively.

All nucleated cells are able to sense and respond to the availability of O_2_[4]. Rat MSCs modify the expression of molecules involved in cell proliferation and survival when they are exposed to low oxygen tensions that approximate physiological conditions [5]. Hypoxia inducible factor 1α (HIF-1α) regulates the expression of many cell cycle molecules, including p21, anti-apoptotic factors, such as Bcl-2 [6], and pro-apoptotic proteins, such as p53 [7]. Consequently, rat MSCs exhibit different proliferation rates when cell expansion under hypoxia and normoxia are compared; however, some controversy exists regarding whether low oxygen tension enhances [8] or suppresses proliferation [9]. Additionally, oxygen plays an important role in the differentiation [10] and maintenance of stemness in MSCs [11].

Due to the inability of tendons and articulations to heal properly, MSC-based therapies have been utilised in horses to treat orthopaedic disorders resulting from sporting endeavours [12,13]. Oxygen levels in cartilage are among the lowest throughout the body [14], and hypoxia appears to be essential for tendon repair [15]. In addition, hypoxic preconditioning improves the therapeutic potential of human MSCs [16]. Taken together, these facts suggest that horse MSCs cultured in hypoxia might constitute a more relevant model for the treatment of injuries in low-oxygen tissues than those currently utilised, which are usually cultured in 20% O_2_.

To improve the methodology for equine stem cell therapy, it is necessary to examine the characteristics and to compare the behaviour of MSCs in normoxic and hypoxic conditions. Specifically, this study contrasts the proliferation kinetics, viability, cell cycle progression, phenotype and stemness of MSCs derived from bone marrow (BM-MSCs) and adipose tissue (AT-MSCs) cultured in 5% and 20% O_2_.

## Results

### Proliferation kinetics

The growth kinetics of BM- and AT-MSCs expanded in normoxia and hypoxia were monitored for 7 days. Normoxic MSCs derived from both sources displayed higher number of cells than hypoxic MSCs at the end of the culture.

**Figure 1:**
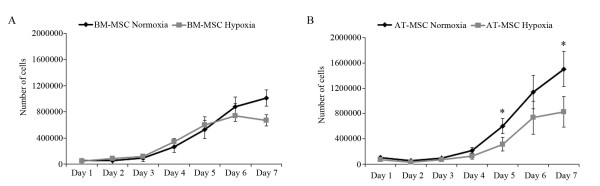
**Growth kinetic curves of equine MSCs at different oxygen concentrations.** Growth kinetics of BM-MSCs (n = 6) (**A**) and AT-MSCs (n = 6) (**B**). The Y axis represents the number of cells, and the X axis represents the number of days in culture. Data are represented as the means ± standard deviation. Black lines correspond to MSCs exposed to 20% O_2_, and grey lines to MSCs exposed to 5% O_2_. (*P < 0.05).

BM-MSCs exposed to both oxygen conditions showed similar lag phase (Figure 1A); however, the log phase lasted less in hypoxic BM-MSCs, until day 5, when they reached a growth plateau state, while normoxic BM-MSCs continued growing slowing down their proliferation the last day of the culture period.

Similarly to BM-MSCs, AT-MSCs at 5% and 20% O_2_ showed similar lag phase and the log phase ended before in hypoxic than in normoxic AT-MSCs, which went on the log phase until the end of the culture period (Figure 1B). Significantly higher number of AT-MSCs in normoxic cultures was detected on days 5 and 7.

### Cell cycle

**Figure 2:**
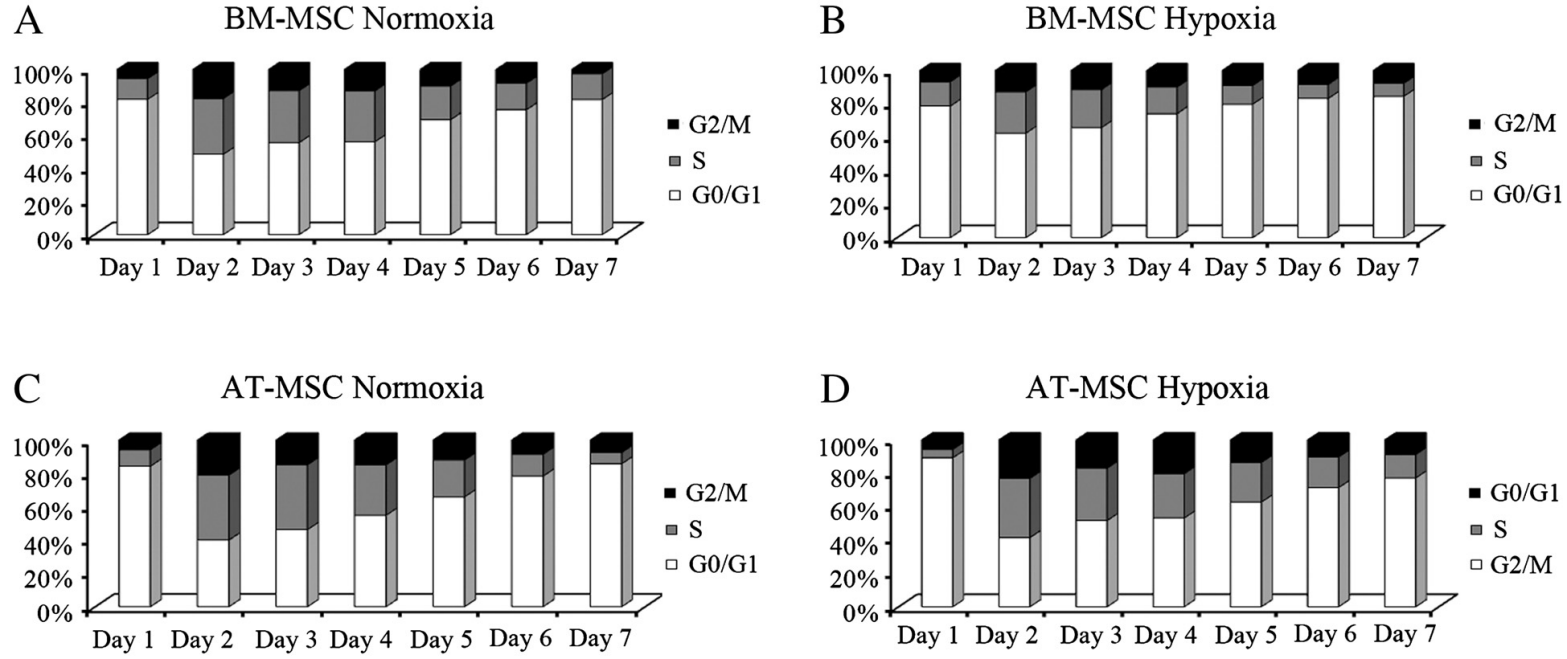
**Cell cycle of equine BM-MSCs and AT-MSCs in normoxic and hypoxic conditions.** Changes of the proportion at cell cycle phases of normoxic BM-MSCs (**A**), hypoxic BM-MSCs (**B**), normoxic AT-MSCs (**C**) and hypoxic AT-MSCs (**D**) for 7 days. Y axis represent the percentage of total cells and the X axis represents the culture days. Black sections represent cells in G_2_/M phases, grey sections represent cells in S phase and white sections represent cells in G_0_/G_1_ phases.

To examine the cell cycle progression under both oxygen conditions, cellular DNA content was quantified in the cultures used in the proliferation study for 7 days. Figure 2 shows the proportions of cells in each cell cycle phase observed in BM- and AT-MSCs expanded in normoxia and hypoxia.

Cell cycle data obtained for BM-MSC cultures showed that normoxic cells were more active than hypoxic cells from day 2 (Table 1)A. Significantly higher percentage of normoxic BM-MSCs was observed in S phase on days 2 and 4, and in G_2_/M phases on day 2. Supporting this finding, significantly higher proportion of hypoxic BM-MSCs in G_0_/G_1_ phases was found on days 2, 3 and 4. However, hypoxic and normoxic AT-MSCs did not display any statistically significant difference over the course of the culture period (Table 1)B.

**Table 1:** Analysis of cell cycle in BM-MSCs (n = 6) and AT-MSCs (n = 6) cultured under hypoxic or normoxic conditions

		**A**		**B**		**C**		**D**	
		**BM-MSC**		**AT-MSC**		**Normoxia**		**Hypoxia**	
		**Normoxia**	**Hypoxia**	**Normoxia**	**Hypoxia**	**BM-MSC**	**AT-MSC**	**BM-MSC**	**AT-MSC**
Day 1	G_0_-G_1_	81.88 ± 15.37	78.64 ± 10.64	84.33 ± 9.48	89.04 ± 5.98	81.88 ± 15.37	84.33 ± 9.48	**78.64 ± 10.64**^**a**^	**89.04 ± 5.98**^**b**^
	S	12.51 ± 10.53	14.51 ± 7.39	9.86 ± 7.17	4.99 ± 3.12	12.51 ± 10.53	9.86 ± 7.17	**14.51 ± 7.39**^**a**^	**4.99 ± 3.12**^**b**^
	G_2_-M	5.61 ± 5.30	6.85 ± 4.70	5.80 ± 2.42	5.74 ± 3.10	5.61 ± 5.30	5.80 ± 2.42	6.85 ± 4.70	5.74 ± 3.10
Day 2	G_0_-G_1_	**49.32 ± 4.46**^**a**^	**62.64 ± 5.52**^**b**^	39.86 ± 3.26	41.33 ± 6.23	**49.32 ± 4.46**^**a**^	**39.86 ± 3.26**^**b**^	**62.64 ± 5.5**^**a**^	**41.33 ± 6.23**^**b**^
	S	**33.93 ± 6.05**^**a**^	**24.51 ± 5.29**^**b**^	39.07 ± 1.50	35.54 ± 7.66	**33.93 ± 6.05**^**a**^	**39.07 ± 1.50**^**b**^	**24.51 ± 5.29**^**a**^	**35.54 ± 7.66**^**b**^
	G_2_-M	**17.89 ± 6.99**^**a**^	**12.85 ± 3.67**^**b**^	21.07 ± 3.52	23.13 ± 2,20	17.89 ± 6.99	21.07 ± 3.52	**12.85 ± 3.67**^**a**^	**23.13 ± 2,20**^**b**^
Day 3	G_0_-G_1_	**56.00 ± 5.85**^**a**^	**65.96 ± 5.83**^**b**^	45.37 ± 3.85	51.57 ± 5.86	**56.00 ± 5.85**^**a**^	**45.37 ± 3.85**^**b**^	**65.96 ± 5.83**^**a**^	**51.57 ± 5.86**^**b**^
	S	31.36 ± 8.24	22.53 ± 5.18	38.36 ± 4.49	31.42 ± 9.07	**31.36 ± 8.24**^**a**^	**38.36 ± 4.49**^**b**^	22.53 ± 5.18	31.42 ± 9.07
	G_2_-M	12.94 ± 4.46	11.50 ± 3.31	14.60 ± 1.04	17.01 ± 6.58	12.94 ± 4.46	14.60 ± 1.04	**11.50 ± 3.31**^**a**^	**17.01 ± 6.58**^**b**^
Day 4	G_0_-G_1_	**56.30 ± 5.65**^**a**^	**74.13 ± 7.11**^**b**^	54.67 ± 2.64	52.74 ± 6.51	56.30 ± 5.65	54.67 ± 2.64	**74.13 ± 7.11**^**a**^	**52.74 ± 6.51**^**b**^
	S	**30.59 ± 10.05**^**a**^	**15.90 ± 6.12**^**b**^	30.24 ± 1.30	26.40 ± 3.42	30.59 ± 10.05	30.24 ± 1.30	**15.90 ± 6.12**^**a**^	**26.40 ± 3.42**^**b**^
	G_2_-M	13.00 ± 5.00	9.97 ± 4.26	14.91 ± 2.34	20.19 ± 8.15	13.00 ± 5.00	14.91 ± 2.34	9.97 ± 4.26	20.19 ± 8.15
Day 5	G_0_-G_1_	69.77 ± 11.77	79.77 ± 7.22	65.83 ± 5.76	62.63 ± 9.25	69.77 ± 11.77	65.83 ± 5.76	**79.77 ± 7.22**^**a**^	**62.63 ± 9.25**^**b**^
	S	20.16 ± 13.11	11.18 ± 4.44	22.18 ± 3.15	23.85 ± 5.48	20.16 ± 13.11	22.18 ± 3.15	**11.18 ± 4.44**^**a**^	**23.85 ± 5.48**^**b**^
	G_2_-M	10.07 ± 2.33	9.06 ± 5.04	11.99 ± 2.75	13.55 ± 4.23	10.07 ± 2.33	11.99 ± 2.75	9.06 ± 5.04	13.55 ± 4.23
Day 6	G_0_-G_1_	75.51 ± 15.79	83.07 ± 6.31	78.36 ± 1.89	71.49 ± 11.70	75.51 ± 15.79	78.36 ± 1.89	83.07 ± 6.31	71.49 ± 11.70
	S	16.08 ± 15.59	8.42 ± 3.02	13.16 ± 1.80	18.43 ± 9.18	16.08 ± 15.59	13.16 ± 1.80	8.42 ± 3.02	18.43 ± 9.18
	G_2_-M	8.36 ± 1.67	8.35 ± 5.52	8.49 ± 1.01	10.35 ± 2.53	8.36 ± 1.67	8.49 ± 1.01	8.35 ± 5.52	10.35 ± 2.53
Day 7	G_0_-G_1_	81.71 ± 6.83	84.50 ± 8.33	85.78 ± 1.56	77.16 ± 12.67	81.71 ± 6.83	85.78 ± 1.56	84.50 ± 8.33	77.16 ± 12.67
	S	15.65 ± 16.21	7.78 ± 4.03	6.73 ± 1.32	14.06 ± 9.66	15.65 ± 16.21	6.73 ± 1.32	7.78 ± 4.03	14.06 ± 9.66
	G_2_-M	2.64 ± 2.01	7.77 ± 4.92	7.50 ± 1.26	8.78 ± 3.20	2.64 ± 2.01	7.50 ± 1.26	7.77 ± 4.92	8.78 ± 3.20

The G_0_/G_1_, S and G_2_/M cell cycle phases were analysed for 7 days. Cell cycle data are represented as the mean ± standard deviation. The “a” and “b” superscripted bold data are significantly different. (A) Compares normoxic and hypoxic conditions of BM-MSCs; (B) compares normoxic and hypoxic conditions of AT-MSCs; (C) compares BM-MSCs and AT-MSCs in normoxic conditions; and (D) compares BM-MSCs and AT-MSCs in hypoxic conditions.

Comparing normoxic cultures of BM-MSCs and AT-MSCs, BM-MSCs displayed a significantly higher percentage of cells in G_0_/G_1_ and reduced frequency of cells in S phase compared with AT-MSCs on days 2 and 3. The proportions of cells in each phase of the cell cycle were comparable throughout the remaining time course (Table 1)C.

Differences in cell cycle progression in hypoxic MSCs derived from both sources were more marked than in normoxia (Table 1)D. On the first day of culture, there were significantly higher percentages of AT-MSCs in G_0_/G_1_ phase and of BM-MSCs in S phase. However, in the following days, until day 5, this behaviour was inverted: significantly more BM-MSC were in G_0_/G_1_, whereas AT-MSCs were more active in cell division and more abundant in S phase on days 2, 4 and 5 and in G_2_/M phase on days 2 and 3.

### Cell viability

**Figure 3:**
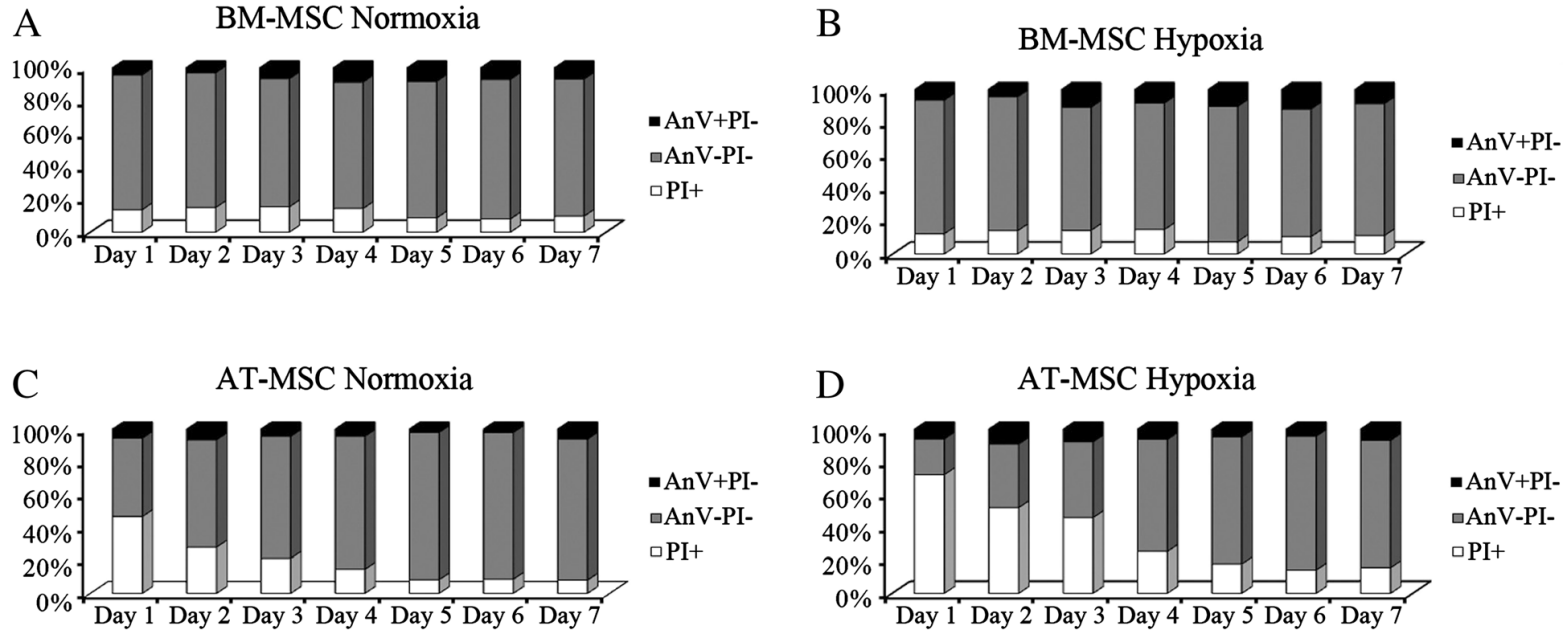
**Viability and apoptosis of equine BM-MSCs and AT-MSCs in normoxic and hypoxic conditions.** Variations in the viability and apoptosis of normoxic BM-MSCs (**A**), hypoxic BM-MSCs (**B**), normoxic AT-MSCs (**C**) and hypoxic AT-MSCs (**D**) for 7 days. Y axis represent the percentage of total cells and the X axis represents the culture days. Black sections represent AnV^+^PI^-^ cells, grey sections represent AnV^-^PI^-^ cells and white sections represent PI^+^ cells.

Possible changes in apoptosis and viability were monitored in the cells used in the proliferation study during 7 days using Annexin V (AnV) and propidium iodide (PI). Figure 3 shows the proportions of viable, apoptotic and non-viable cells in the different cultures during the culture period.

**Figure 4:**
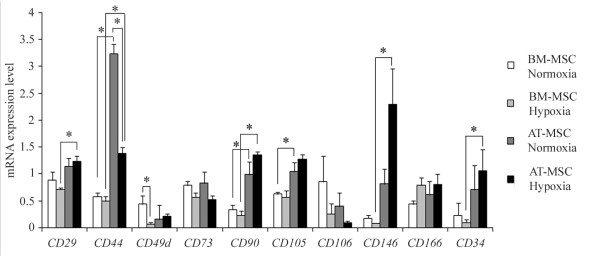
**Gene expression of *CD29, CD34, CD44, CD49d, CD73, CD90, CD105, CD106, CD146 *and *CD166 *cell surface markers of equine MSCs.** Relative mRNA expression levels are expressed as the mean ± standard error. White bars correspond to normoxic BM-MSCs (n = 6), light grey bars with hypoxic BM-MSCs (n = 6), dark grey bars with normoxic AT-MSCs (n = 6) and black bars with hypoxic AT-MSCs (n = 6). (*P < 0.05).

**Figure 5:**
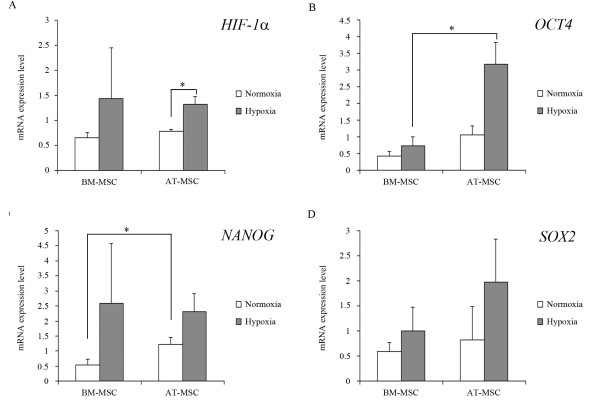
**Gene expression of the pluripotency markers in equine MSCs.** Relative mRNA expression levels are expressed as the mean ± standard error for ***HIF-1α*** (**A**), *OCT4* (**B**), *NANOG* (**C**) and *SOX2* (**D**) . White bars correspond to MSCs cultured under normoxia (n = 6), and grey bars correspond to MSCs cultured under hypoxia (n = 6). *P < 0.05.

BM-MSCs expanded in both oxygen atmospheres showed similar proportions of apoptotic (AnV^+^ PI^-^) and non-viable cells (PI^+^) during the 7 days of culture (Table 2)A. However, on culture days 1, 2, 3 and 5, a significantly higher proportion of viable cells was observed in normoxic AT-MSCs than in hypoxic AT-MSCs (Table 2)B. These differences were associated with a significantly higher frequency of PI^+^ cells in hypoxic conditions on days 3, 4, 5 and 7, while the proportion of AnV^+^ PI^-^ cells was similar for both conditions and never higher than 10% of the total population.

**Table 2:** Viability of BM-MSCs (n = 6) and AT-MSCs (n = 6) cultured under hypoxic or normoxic conditions

		**A**		**B**		**C**		**D**	
		**BM-MSC**		**AT-MSC**		**Normoxia**		**Hypoxia**	
		**Normoxia**	**Hypoxia**	**Normoxia**	**Hypoxia**	**BM-MSC**	**AT-MSC**	**BM-MSC**	**AT-MSC**
Day 1	PI^+^	13.64 ± 2,03	12.06 ± 3.89	46.64 ± 26.38	72.24 ± 17.20	**13.64 ± 2,03**^**a**^	**46.64 ± 26.38**^**b**^	**12.06 ± 3.89**^**a**^	**72.24 ± 17.20**^**b**^
	AnV^-^PI^-^	81.92 ± 2.74	81.39 ± 8.51	**47.86 ± 23.61**^**a**^	**21.79 ± 12.35**^**b**^	**81.92 ± 2.74**^**a**^	**47.86 ± 23.61**^**b**^	**81.39 ± 8.51**^**a**^	**21.79 ± 12.35**^**b**^
	AnV^+^PI^-^	4.44 ± 1.27	6.52 ± 4.94	5.60 ± 2.69	6.05 ± 6.04	4.44 ± 1.27	5.60 ± 2.69	6.52 ± 4.94	6.05 ± 6.04
Day 2	PI^+^	15,02 ± 3.05	13,97 ± 6.37	28.01 ± 11.11	52.85 ± 25.16	**15,02 ± 3.05**^**a**^	**28.01 ± 11.11**^**b**^	**13,97 ± 6.37**^**a**^	**52.85 ± 25.16**^**b**^
	AnV^-^PI^-^	81.91 ± 3.67	81.72 ± 7.00	**65.45 ± 12.05**^**a**^	**39.31 ± 22.23**^**b**^	**81.91 ± 3.67**^**a**^	**65.45 ± 12.05**^**b**^	**81.72 ± 7.00**^**a**^	**39.31 ± 22.23**^**b**^
	AnV^+^PI^-^	3.07 ± 0.94	4.53 ± 1.52	6.54 ± 2.53	9.31 ± 5.23	**3.07 ± 0.94**^**a**^	**6.54 ± 2.53**^**b**^	4.53 ± 1.52	9.31 ± 5.23
Day 3	PI^+^	15.44 ± 7.98	14.07 ± 5.08	**21,29 ± 3.94**^**a**^	**45.97 ± 19.78**^**b**^	15.44 ± 7.98	21,29 ± 3.94	**14.07 ± 5.08**^**a**^	**45.97 ± 19.78**^**b**^
	AnV^-^PI^-^	77.84 ± 3.96	75.04 ± 11.65	**74.28 ± 3.13**^**a**^	**46.24 ± 18.6**^**b**^	**77.84 ± 3.96**^**a**^	**74.28 ± 3.13**^**b**^	**75.04 ± 11.65**^**a**^	**46.24 ± 18.61**^**b**^
	AnV^+^PI^-^	6.71 ± 2.16	10.86 ± 7.19	4.43 ± 1.62	7.83 ± 5.23	6.71 ± 2.16	4.43 ± 1.62	10.86 ± 7.19	7.83 ± 5.23
Day 4	PI^+^	14.43 ± 6.25	14.60 ± 7.41	**14.64 ± 3.60**^**a**^	**25.39 ± 11.67**^**b**^	14.43 ± 6.25	14.64 ± 3.60	**14.60 ± 7.41**^**a**^	**25.39 ± 11.67**^**b**^
	AnV^-^PI^-^	76.82 ± 5.89	77.11 ± 7.49	80.98 ± 2.88	68.31 ± 11.32	76.82 ± 5.89	80.98 ± 2.88	77.11 ± 7.49	68.31 ± 11.32
	AnV^+^PI^-^	8.74 ± 4.06	8.31 ± 3.15	4.41 ± 2.40	6.30 ± 5.49	**8.74 ± 4.06**^**a**^	**4.41 ± 2.40**^**b**^	8.31 ± 3.15	6.30 ± 5.49
Day 5	PI^+^	8.7 ±3.27	7.34 ± 2.61	**8.29 ± 1.80**^**a**^	**17.82 ± 7.72**^**b**^	8.7 ±3.27	8.29 ± 1.80	**7.34 ± 2.61**^**a**^	**17.82 ± 7.72**^**b**^
	AnV^-^PI^-^	83.03 ± 4.91	82.28 ± 8.29	**89.64 ± 1.54**^**a**^	**77.37 ± 6.67**^**b**^	**83.03 ± 4.91**^**a**^	**89.64 ± 1.54**^**b**^	**82.28 ± 8.29**^**a**^	**77.37 ± 6.67**^**b**^
	AnV^+^PI^-^	8.28 ± 3.49	10.37 ± 7.60	2.06 ± 0.73	4.81 ± 4.22	**8.28 ± 3.49**^**a**^	**2.06 ± 0.73**^**b**^	10.37 ± 7.60	4.81 ± 4.22
Day 6	PI^+^	8.04 ± 4.37	10.27 ± 1.59	8.64 ± 1.51	14.14 ± 8,83	8.04 ± 4.37	8.64 ± 1.51	10.27 ± 1.59	14.14 ± 8,83
	AnV^-^PI^-^	84.79 ± 7.37	77.76 ± 9.44	89.18 ± 1.32	81.43 ± 10.06	84.79 ± 7.37	89.18 ± 1.32	77.76 ± 9.44	81.43 ± 10.06
	AnV^+^PI^-^	7.16 ± 3.46	11.98 ± 7.96	2.14 ± 0.66	4.42 ± 2.71	**7.16 ± 3.46**^**a**^	**2.14 ± 0.66**^**b**^	11.98 ± 7.96	4.42 ± 2.71
Day 7	PI^+^	9.77 ± 5,27	10.95 ± 4.29	**8.05 ± 4.28**^**a**^	**15.69 ± 8.96**^**b**^	9.77 ± 5,27	8.05 ± 4.28	10.95 ± 4.29	15.69 ± 8.96
	AnV^-^PI^-^	83.29 ± 6.91	80.45 ± 5.98	85.67 ± 7.91	78.05 ± 7.77	83.29 ± 6.91	85.67 ± 7.91	80.45 ± 5.98	78.05 ± 7.77
	AnV^+^PI^-^	6.92 ± 3.40	8.65 ± 7.26	6.14 ± 4.32	6.96 ± 1.16	6.92 ± 3.40	6.14 ± 4.32	8.65 ± 7.26	6.96 ± 1.16

Viability data are represented as the mean ± standard deviation. Non-viable cells (PI^+^), viable cells (AnV^-^PI^-^) and apoptotic cells (AnV^+^PI^-^) were analysed for 7 days. Superscripted bold data (“a” and “b”) are significantly different. (A) Compares normoxic and hypoxic conditions of BM-MSCs; (B) compares normoxic and hypoxic conditions of AT-MSCs; (C) compares BM-MSCs and AT-MSCs in normoxic conditions; and (D) compares BM-MSCs and AT-MSCs in hypoxic conditions.

Comparing the viability of normoxic BM-MSC and AT-MSC culture (Table 2)C, the proportions of viable BM-MSCs were significantly higher than in AT-MSCs until day 3. Since then this trend reversed, being the proportion of viable cells significantly higher in AT-MSCs than in BM-MSCs on days 4 and 5. The differences in viability between both cell types observed at early stages of culture resulted from a significantly higher proportion of non-viable AT-MSCs on days 1 and 2; as the time of culture went by, the percentage of PI^+^ AT-MSCs became similar and the proportion of apoptotic BM-MSCs increased, being significantly higher on days 2, 4, 5 and 6.

The behaviour displayed by BM-MSCs and AT-MSCs in hypoxia was similar to their normoxic equivalents until day 4 (i.e., significantly higher percentages of PI^+^ and lower percentages of AnV^-^PI^-^ cells were detected in AT-MSC cultures). However, unlike normoxic MSCs, these results were maintained until the end of the culture period. In addition, the proportion of AnV^+^PI^-^ was similar for both cell types, which contrasts with the results of comparisons between normoxic MSCs (Table 2)D.

### Immunophenotype and gene expression patterns of surface markers

The immunophenotype for the surface markers CD29 and CD90 was analysed using flow cytometry, which revealed similar expression patterns for the MSCs independently of source or oxygen atmosphere (Table 3). In all cases, the percentage of positive cells was greater than 93%.

**Table 3:** Immunophenotype of BM-MSCs and AT-MSCs cultured under normoxic and hypoxic conditions

	**CD29**	**CD90**
BM-MSC Normoxia	99.73 ± 0.03	97.40 ± 0.66
BM-MSC Hypoxia	99.41 ± 0.26	98.06 ± 0.60
AT-MSC Normoxia	97.93 ± 0.83	96.52 ± 1.01
AT-MSC Hypoxia	98.75 ± 1.33	93.65 ± 3.92

Data are expressed as the mean ± standard deviation of percentages of MSCs positive for CD29 and CD90 expression. The expression data were obtained using flow cytometry.

**Table 4:** Summary of gene information

**Gene**	**Accession number**	**Primer sequence (5’-3’)**	**Amplicon size (bp)**
*CD29*^a^	XM_001492665	F: GTAAAAAGTCTTGGAACCGATCTGAT	81
		R: CACAAATGAGCCAAACCCAATT	
*CD34*^a^	XM_001491596	F:CACTAAACCCTCTACATCATTTTCTCCTA	150
		R: GGCAGATACCTTGAGTCAATTTCA	
*CD44*^a^	NM_001085435	F: CCCACGGATCTGAAACAAGTG	95
		R: TTCTGGAATTTGAGGTCTCCGTAT	
*CD49d*^a^	XM_001917601	F: CATCGGCCTTCTCACAGAGAA	101
		R: GCCATTATTGTCTGCATCAATTTG	
*CD73*^a^	XM_001500115	F: GGGATTGTTGGATACACTTCAAAAG	90
		R: GCTGCAACGCAGTGATTTCA	
*CD90*^a^	EU881920	F: TGCGAACTCCGCCTCTCT	93
		R: GCTTATGCCCTCGCACTTG	
*CD105*^a^	XM_001500078	F: GACGGAAAATGTGGTCAGTAATGA	100
		R: GCGAGAGGCTCTCCGTGTT	
*CD106*^a^	NM_001101650	F: CATCGTGACCTGTGGGCATA	111
		R: TGGGTTTCCCTCCACTAGCA	
*CD146*^a^	XM_001917594	F: CTGGACTTGGAAACCACAACATC	85
		R: CAGGTCTCACTCGGACATCAGA	
*CD166*^a^	XM_001503380	F: GTCTGGTCTTCTGCCTCTTGATC	103
		R: TCGGCAAGGCATGATAATAGTG	
*HIF-1α*	XM_001493206	F: AATCCAAAGATCCTGGCGTTG	103
		R: GCTGCTGTAGTAATGCGCCAAT	
*OCT4*^b^	XM_001490108	F: AGAGGCAACCTGGAGAACATG	70
		R: GGGCAATGTGGCTGATCTG	
*NANOG*^b^	XM_001498808	F: TACCTCAGCCTCCAGCAGAT	119
		R: CAGTTGTTTTTCTGCCACCT	
*SOX2*^c^	FJ356148	F: TGGTTACCTCTTCCTCCCACT	178
		R: GGGCAGTGTGCCGTTAAT	
*GAPDH*^a^	NM_001163856	F: GGCAAGTTCCATGGCACAGT	128
		R: CACAACATATTCAGCACCAGCAT	
*B2M*^a^	NM_001082502	F: TCGTCCTGCTCGGGCTACT	102
		R: ATTCTCTGCTGGGTGACGTGA	

^a^ previously described in [43].

^b^ previously described in [40].

^c^ previously described in [47].

Genes analysed, GenBank accession numbers, primer sequences for reverse transcriptase RT-PCR (F: forward and R: reverse) and amplicon sizes in base pairs (bp).

In addition to flow cytometry, real time quantitative PCR (RT-qPCR) was performed to assess the expression of *CD29* and *CD90,* as well as 8 additional surface antigens (Figure 4). Few significant differences in gene expression were found between cells from the same origin that were expanded in different oxygen conditions. The expression of the *CD49d* gene was significantly higher in normoxic BM-MSCs than in their hypoxic counterparts. In AT-MSCs, *CD44* expression was significantly higher in normoxia.

Further differences in gene expression were observed when cultures from different sources exposed to the same oxygen tension were compared. In general, there was a trend of higher gene expression for all surface markers analysed in AT-MSCs, being statistically significant for *CD44*, *CD90* and *CD105* in normoxia and *CD44*, *CD29*, *CD34*, *CD90* and *CD146* in hypoxia. A tendency of a higher expression in BM-MSCs was detected for *CD49d* in normoxia, and *CD106* in both normoxic and hypoxic conditions. Similar gene expression patterns of the surface antigens *CD73* and *CD166* were detected in the four conditions (two types of cells grown under two oxygen treatments). Although the level of *CD106* mRNA was very low in hypoxic AT-MSCs, any significant differences existed between the two tissue sources and oxygen conditions.

### Hipoxia inducible factor 1α and pluripotency markers

The gene expression of *HIF-1α* and pluripotency markers was measured in both normoxic and hypoxic cells using RT-qPCR.

Gene expression of *HIF-1α* was detected in BM-MSCs and AT-MSCs in both oxygen conditions (Figure 5A). The mRNA levels were higher in hypoxic MSCs than in normoxic MSCs derived from the two sources, being statistically significant for AT-MSCs. BM-MSCs and AT-MSCs expressed similar levels of *HIF-1α* for each oxygen condition.

Transcripts of the embryonic stem cell makers *OCT4*, *NANOG* and *SOX2* were detected in BM-MSCs and AT-MSCs expanded in both oxygen conditions (Figure 5B-D). The mRNA levels were consistently higher in AT-MSCs than in BM-MSCs, with statistically significant differences for the gene expression of *OCT4* in hypoxia and *NANOG* in normoxia.

The MSCs exposed to 5% O_2_ showed a tendency to express higher levels of the three genes than the MSCs exposed to 20% O_2_.

## Discussion

In the equine veterinary field, orthopaedic injuries are a major cause of retirement of athletic horses [17]. As a result, it is not surprising that equine regenerative medicine is primarily focused on the treatment of musculoskeletal defects. The present cell therapy studies are carried out with MSCs [12,13,18] and non-adult stem cells [19-21]. To better understand the mechanisms of action of MSCs *in vivo*, a large number of studies to characterise equine MSCs have been reported over the last five years [22-26]. However, because the overall objective of regenerative treatments is the use of MSCs in live horses, it is important to determine all of the properties of MSCs in an oxygen environment that closely emulates the original physiological niche from which the cells derive. To our knowledge, the current work constitutes the first study to perform an analysis of the influence of oxygen tension on proliferation, viability, stemness and marker expression in equine MSCs derived from bone marrow and adipose tissue.

The effects of hypoxia on MSC proliferation have been studied specifically in humans and mice. Enhancements in cell growth following exposure to hypoxia have been described [10,11,27]. However, there is no unanimous consent, Feher et al. (2010) reported no difference in the growth of normoxic and hypoxic cells, and Volker et al. (2010) described similar numbers of cells for both oxygen conditions at the conclusion of the culture period. In addition, Holzwarth et al. (2010), Zeng et al. (2011) and Wang et al. (2005) reported that low oxygen tension inhibited the proliferation of MSCs. Similarly, canine MSCs derived from bone marrow and adipose tissue exposed to atmospheric O_2_ show more proliferative capacity than those expanded from passage 1 to passage 3 under hypoxic conditions (1% or 5% O_2_) [28]. In agreement with these findings, our results describing the proliferation of equine cells as a function of oxygen tension showed that the growth of AT-MSCs was significantly higher at atmospheric oxygen tension, while BM-MSCs underwent also more proliferation in 20% O_2_.

Differences in cell growth between cultures expanded under different oxygen conditions could result from cell cycle changes or alterations of cell viability. Human MSC populations derived from umbilical cord and bone marrow accumulate cells in G_0_/G_1_ phase under low oxygen tension [9,29]. Similar to these experiments, we found that hypoxic BM-MSCs displayed a higher percentage of cells in G_0_/G_1_ phases than normoxic BM-MSCs throughout the entire culture period. Moreover, the significantly higher proportion of normoxic BM-MSCs involved in the active stages of cell division (S or G_2_/M) during the median days of culture led to a higher number of BM-MSCs at the conclusion of proliferation assay in the normoxic culture. Cellular arrest in G_0_/G_1_ phase in hypoxic BM-MSCs might be caused by up-regulation of cyclin-dependent kinase inhibitors that control the cell cycle checkpoint [30-32].

In contrast to BM-MSCs, differences observed in the proliferation of normoxic and hypoxic AT-MSC cultures were not due to cell cycle variations, but to variations in cell viability. Similarly to rat MSCs, that undergo a reduction in cell viability when permanently exposed to hypoxia [33], in our work the proportions of viable AT-MSCs in hypoxic cultures were always lower than those in normoxic cultures. Reduced viability in hypoxic conditions reflects insufficient adaptation of AT-MSCs at 5% O_2_, as higher percentages of non-viable cells were found in hypoxic conditions relative to populations at 20% O_2_. No detectable changes in apoptosis have been previously described for hypoxic MSCs [34,35]; our results corroborate these reports since the proportion of AnV^+^PI^-^ did not display statistical differences between normoxic and hypoxic MSCs derived from the same source.

Moreover, AT-MSCs under either oxygen tension adapted more poorly to the culture environment following trypsinisation than BM-MSCs, as shown by a significantly higher proportion of PI^+^ AT-MSCs at days 1 and 2 for both 5% and 20% O_2_ atmospheres. This is reflected in the increased lag phase displayed by AT-MSCs. However, AT-MSCs also showed a significantly increased proportion of cells undergoing cell division during the first days of culture. The increase in cell division of viable AT-MSCs might compensate for cell death in the population because the final number of cells obtained at the end of the experiment was higher in AT-MSC cultures than in BM-MSC cultures, which indicates a higher proliferative ability for AT-MSCs than BM-MSCs. This result is in agreement with previous reports in horses [26] and other species as canine [28], rat [36] and human [37], which demonstrated AT-MSCs proliferated more rapidly than BM-MSC. In our experimental conditions, AT-MSCs in normoxic condition did not display a plateau phase in the proliferation curve and, at the light microscope, AT-MSCs start growing in several layers instead of an only monolayer (data not shown). These observations might point out a lack of contact inhibition of growth in AT-MSCs. In addition, in a previous study, we described the more rapid decrease of apoptosis in AT-MSCs compared with BM-MSCs in cultures at 20% O_2_ using a limited number of animals (n = 2) [26]. The current study confirms that finding because normoxic AT-MSCs showed significantly lower proportion of apoptotic cells than normoxic BM-MSCs.

Flow cytometric immunophenotype analysis of horse MSCs revealed that the surface antigen CD90 was detectable in all MSC types [38-41]. In addition, cross-reactivity with human antibodies has been demonstrated for the CD29 antigen in a previous report from our group and also in other studies [42,43]. Because in other species hypoxia does not alter the immunophenotype of MSCs with regard to CD29 [29] and CD90 [9,27,44], we attempted to characterise this phenotype in equine MSCs and to analyse the presence of these molecules in both BM-MSCs and AT-MSCs in hypoxia and normoxia. According to the literature, equine MSCs displayed the same immunophenotype for CD29 and CD90 independently of the cell source and oxygen tension.

The lack of immunoreactivity of commercial antibodies with equine MSC antigens remains a challenge in determining the immunophenotype of these cells by flow cytometry. As a supplement to this technique, RT-qPCR has been used to establish the expression profiles of various cell surface markers in equine MSCs [39,42]. Similar gene expression patterns were demonstrated in AT-MSCs when they were compared to BM-MSCs in their respective oxygen conditions. AT-MSCs at both oxygen tensions expressed higher levels of *CD29*, *CD44*, *CD90*, *CD146* and *CD34* transcripts respect to BM-MSCs; in contrast, only normoxic AT-MSCs expressed lower mRNA levels of *CD49d* compared to normoxic BM-MSCs. These results are in agreement with our previous report [43]. The differences in *CD105* expression, with respect to our previous work, might be due to individual differences because different animals were used in the present study. Hypoxia seemed to significantly modify mRNA levels of *CD49d* in BM-MSCs and *CD44* in AT-MSCs, which is in agreement with other studies that have described different expression profiles for *CD49d*[45] and *CD44*[46] in hypoxia. The remaining surface markers analysed in this study showed similar gene expression pattern at the different oxygen conditions studied.

HIF-1α is a transcription factor that is expressed constitutively in cells, although is ubiquitinated and degraded under normoxic conditions. In our study, the gene expression of this factor was detected in normoxic and hypoxic cultures, although *HIF-1α* was up-regulated in cultures exposed to low oxygen tension.

The expression of specific markers characteristic of embryonic stem cells have been described before for equine BM-MSCs [47] and AT-MSCs [40]. However, to our knowledge, this is the first work that compares the gene expression of the pluripotency markers *OCT4*, *NANOG* and *SOX2* in equine AT-MSCs and BM-MSCs that were exposed to different oxygen concentrations. In our experimental conditions, equine MSCs expressed all three pluripotency markers. In general, higher expression of each marker was detected in AT-MSCs and was statistically significant for *OCT4* in hypoxia and for *NANOG* in normoxia. The consistently higher expression of all genes in hypoxia might reflect the enhanced stemness of hypoxic equine MSCs [48]. These results agree with other studies that have described up-regulation of pluripotency-associated markers of hypoxic MSCs [11,49,50]. To our knowledge the relationship between pluripotency markers and HIF-1α has never been investigated in MSCs. However, studies in cancer cells have revealed the expression of HIF-1α induces a gene expression increase of genes involved in stemness [51]; in accordance with this, the higher expression of *HIF-1α* observed in hypoxic cultures of equine MSCs could enhance the gene expression of the pluripotency markers. Taken together, the results might suggest that low oxygen tension helps maintain the undifferentiated stem cell phenotype.

## Conclusions

Oxygen plays a deterministic role in equine MSC cultures. It is able to modify their proliferative capacity via cell cycle modification in BM-MSCs and alterations in cell viability in AT-MSCs. Moreover, the immunophenotype of both MSC types is not altered by hypoxia. However, hypoxia appears to be an important factor in the maintenance or acquisition of stemness in equine MSCs.

## Methods

### Animals

In order to work with a homogeneous group of animals to reduce the interindividual differences derived from age, breed or sex, biological samples were obtained from a total of 12 castrated male horses aged from 4 to 7 years. All procedures were carried out under Project Licence PI36/07, which was approved by the Ethic Committee for Animal Experiments from the University of Zaragoza. The care and use of animals were performed in accordance with the Spanish Policy for Animal Protection RD1201/05, which meets the European Union Directive 86/609 on the protection of animals for experimental and other scientific purposes.

### MSC isolation, culture and expansion

Samples were collected as previously described [26,43]. Briefly, bone marrow aspirates were harvested from the sternum of six horses. The mononuclear fractions were enriched with MSCs, which were isolated in a centrifugation gradient using Lymphoprep (Atom, Barcelona, Spain). Isolated MSCs were rinsed twice with PBS and plated at a concentration of 10^6^ cells/cm^2^ in growth medium, which consisted of DMEM Low Glucose (Sigma-Aldrich, St. Louis, Missouri, USA) supplemented with foetal bovine serum, L-glutamine (Sigma-Aldrich, St. Louis, Missouri, USA) and penicillin/streptomycin (Sigma-Aldrich, St. Louis, Missouri, USA).

Subcutaneous adipose tissues were collected from the dorsal gluteal muscle below the tail from six horses. The stromal vascular fractions (SVFs) were isolated by digestion with 0.01% collagenase (Type I, Sigma-Aldrich, St. Louis, Missouri, USA) for 30 min at 37°C with continuous shaking. The cells were rinsed twice with PBS and plated in growth medium at a concentration of 10^5^ cells/cm^2^.

Both MSC types were expanded for 4 weeks at 37°C in either normoxic (5% CO_2_ and 20% O_2_) or hypoxic (5% CO_2_ and 5% O_2_) conditions. Cell growth kinetics, immunophenotype, viability and cell cycle analyses were carried out using newly passaged cells fresh cells. Aliquots of 10^6^ cells were preserved at −150°C at the final passage, as described above, for further gene expression studies.

### Cell growth kinetics

Cells from bone marrow and adipose tissue were seeded in 6-well plates in triplicate at a density of 5,000 cells/cm^2^. BM-MSCs (n = 6) and AT-MSCs (n = 6) were exposed to 20% O_2_ or 5% O_2_ atmospheres for 7 days. Every day, the cells were collected using 0.25% trypsin/EDTA, and an aliquot of 50 μL of each culture was counted in a haemocytometer Z2 Coulter particle count and size analyser to obtain growth curves.

### Analysis of cellular DNA content

Half of the MSCs harvested from the proliferation assay were fixed in 70% ice-cold ethanol and treated with 0.02 mg/mL RNAse and EDTA. DNA was stained with 0.1 mg/mL propidium iodide (Sigma-Aldrich, St. Louis, Missouri, USA). Cells were incubated in the dark for 30 min, and samples were analysed on a FACSARRAY (BD Biosciences, East Rutherford, New Jersey, USA) cytometer using the MODIFIT 3.0 software.

### Viability assay

The remaining fraction of MSCs harvested in the proliferation assay was used to determine MSC viability. Apoptosis was measured by the detection of phosphatidylserine on the outer leaflet of the plasma membrane with the fluorescent dye Annexin V-FITC (Immunostep, Salamanca, Spain) in accordance with the manufacturer’s instructions. Briefly, cells were rinsed with ice-cold PBS and then resuspended in 200 μL of binding buffer. Subsequently, 10 μL of Annexin V stock solution was added to cells and incubated for 30 min at 4°C. Non-viable cells were identified by incubation with 5 μL of propidium iodide, a dye that penetrates into the cell nucleus when the plasma and nuclear cell membranes are damaged. PI-stained cells were immediately analysed in a FACSARIA cytometer (BD Biosciences, East Rutherford, New Jersey, USA) using FACSDIVA 5.0.1 software.

### Immunophenotyping

To determine the immunophenotype of BM-MSCs and AT-MSCs after hypoxic and normoxic culture, the expression of the MSC surface markers CD29 (Integrin β1) and CD90 (Thy-1) was assessed by flow cytometry as previously described [43] using mouse anti-human monoclonal antibodies CD29-FITC (Caltag Laboratories, Little Balmer, Buckingham, UK) and CD90-PE (BD Pharmingen, San Diego, California, USA.). Negative control staining was performed using a FITC-conjugated mouse IgG1 isotype and a PE-conjugated mouse isotype. The immunophenotype was determined with the cytometer and software described above.

### Gene expression analysis

The expression of 10 genes encoding cell surface molecules, including *CD29* and *CD90*, was determined by real-time quantitative PCR. Additional antigens examined were *CD34*, *CD44* (H-CAM), *CD49d* (α4 integrin), *CD73* (ecto-5’-nuclease), *CD105* (endoglin), *CD106* (VCAM 1), *CD146* (MCAM) and *CD166* (ALCAM). The gene expression levels of the pluripotency markers *OCT4*, *SOX2* and *NANOG* were also analysed using the same technique.

Total RNA was extracted using the RNA spin mini (GE Healthcare Lifesciences, Little Chalfont, UK) and DNAse turbo (Ambion, Foster City, California, USA.) kits; subsequently, the Superscript kit (Invitrogen, Carlsbad, CA, USA ) was used for reverse transcription of 1.5 μg of total RNA into complementary DNA. All kits were used in accordance with the manufacturer’s instructions.

Table 4 shows the names of the analysed genes, GenBank accession numbers for equine mRNA sequences, forward and reverse primer sequences and amplicon sizes. Amplifications were performed in triplicate using the Fast SYBR Green Master Mix reagent (Applied Biosystems, Foster City, California, USA) and the StepOne™ Real Time System (Applied Biosystems, Foster City, California, USA). The levels of gene expression were determined using the comparative Ct method. A normalisation factor was calculated as the geometric mean of the quantity of two housekeeping genes (*GAPDH* and *B2M*) and used to normalise the expression of each gene.

### Statistical analyses

The software SPSS 19.0 (Armonk, Nueva York, USA) was used to perform statistical analyses. Data obtained from flow cytometry and RT-qPCR were analysed for normality with the Shapiro-Wilk test. Differences in gene expression and reactivity levels in BM- and AT-MSCs expanded under hypoxia and normoxia conditions were determined using unpaired non-parametric Mann–Whitney tests. Differences in proliferation, viability and cell cycle were evaluated with Student’s *t*-test. For both tests, *P <* 0.05 was considered statistically significant.

## Abbreviations

MSC: Mesenchymal stem cell; BM-MSC: Bone marrow-derived mesenchymal stem cell; AT-MSC: Adipose tissue-derived mesenchymal stem cell; AnV: Annexin V; PI: Propidium iodide; AnV^+^PI^-^: Apoptotic cells; PI^+^: Non-viable cells; AnV^-^PI^-^: Viable cells; RT-qPCR: Real time quantitative PCR.

## Competing interests

The authors declare that they have no competing interests.

## Authors’ contributions

BR carried out the expansion of the cells, proliferation assays, gene expression analyses, statistical analysis and drafted the manuscript. ARR participated in the expansion of the cells and proliferation assays. SAA participated in the gene expression analyses. AR performed the sample collections from the horses. FJV participated in the sample collections from the horses. PZ helped to draft the manuscript. IMB conceived the study, participated in its design and helped to draft the manuscript. CR conceived the study, participated in its design and helped to draft the manuscript. All authors read and approved the final manuscript.
